# Effect of calcification on the mechanical stability of plaque based on a three-dimensional carotid bifurcation model

**DOI:** 10.1186/1471-2261-12-7

**Published:** 2012-02-15

**Authors:** Kelvin KL Wong, Pongpat Thavornpattanapong, Sherman CP Cheung, Zhonghua Sun, Jiyuan Tu

**Affiliations:** 1 School of Aerospace, Mechanical and Manufacturing Engineering, and Health Innovations Research Institute (HIRi), RMIT University, Australia; 2 Discipline of Medical Imaging, Department of Imaging and Applied Physics, Curtin University, Australia

**Keywords:** atherosclerosis, calcification, fibrous cap, lipids, plaque rupture

## Abstract

**Background:**

This study characterizes the distribution and components of plaque structure by presenting a three-dimensional blood-vessel modelling with the aim of determining mechanical properties due to the effect of lipid core and calcification within a plaque. Numerical simulation has been used to answer how cap thickness and calcium distribution in lipids influence the biomechanical stress on the plaque.

**Method:**

Modelling atherosclerotic plaque based on structural analysis confirms the rationale for plaque mechanical examination and the feasibility of our simulation model. Meaningful validation of predictions from modelled atherosclerotic plaque model typically requires examination of bona fide atherosclerotic lesions. To analyze a more accurate plaque rupture, fluid-structure interaction is applied to three-dimensional blood-vessel carotid bifurcation modelling. A patient-specific pressure variation is applied onto the plaque to influence its vulnerability.

**Results:**

Modelling of the human atherosclerotic artery with varying degrees of lipid core elasticity, fibrous cap thickness and calcification gap, which is defined as the distance between the fibrous cap and calcification agglomerate, form the basis of our rupture analysis. Finite element analysis shows that the calcification gap should be conservatively smaller than its threshold to maintain plaque stability. The results add new mechanistic insights and methodologically sound data to investigate plaque rupture mechanics.

**Conclusion:**

Structural analysis using a three-dimensional calcified model represents a more realistic simulation of late-stage atherosclerotic plaque. We also demonstrate that increases of calcium content that is coupled with a decrease in lipid core volume can stabilize plaque structurally.

## 1. Background

Atherosclerosis constitutes a high number of deaths related to cardiovascular diseases in developed countries. It is a chronic systemic disease, frequently leading to vascular morbidity and premature mortality. Although atherosclerosis is systemic, plaque rupture is local and leads to acute cardiac syndromes such as ischemia and myocardial infarction or cerebrovascular events. Plaque material and structural characteristics are important factors in the natural progression of the disease and may have important clinical predictive value.

Extensively calcified lesions most likely represent atherosclerosis at later stages of remodelling and may reflect more stable lesions [1]. However, earlier stages of atherosclerosis that do not contain calcium deposits may be more prone to rupture with subsequent occurrence of acute events [2]. Not only can non- or less-invasive imaging identify flow-limiting coronary stenosis [3], but it can also to detect plaque components, measure atherosclerotic plaque burden and its response to treatment, and to differentiate stable plaques from those that are prone to rupture [4,5]. Non-invasive imaging modalities such as computed tomography [6] and magnetic resonance imaging [7-9], as well as the invasive intravascular ultrasound modality [10-12], allow for detection of plaque morphology and composition (calcified versus non-calcified atherosclerotic plaques) and assessment of the extent of remodelling [13].

**Figure 1:**
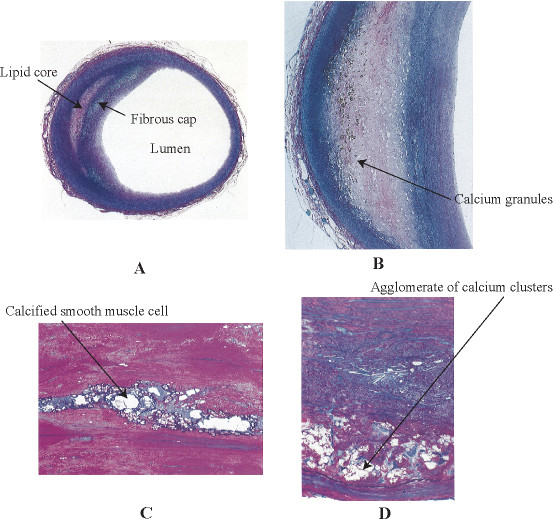
**Histological observation of plaque composites**. A: Lipid distribution in a crescent formation within the plaque can cause protrusion into the lumen and tend to obstruct distal arteries after plaque rupture. B: Calcification can be observed by white granules embedded in lipid. C: A zoom-in view of calcified smooth muscles cells reveals the agglomerate of calcium clusters within the plaque. D: Calcification agglomerates are also present in the lesion adjacent to the elastic lamina. (Revised from images by Stary [14])

Plaques can be characterized into three types based on the histology analysis [14]: non-calcified plaques; calcified plaques; and mixed plaques refer to lesions with non-calcified and calcified components within a single lesion (Figure 1A and 1B). The presence of calcification in lipid, based on observation agglomerate of calcium clusters, occurs in some plaques (Figure 1C and 1D). Calcium content is reported to increase in patients with acute coronary syndrome [15]. Large lipid core and calcified areas (defined as > 10% of the plaque area each) and thin-cap fibroatheroma have been found to be associated with positive vascular remodeling [16,17]. Regardless of the mechanisms of calcium formation involved, histology shows that calcium is a common but variable component in advanced atherosclerotic plaques.

Both composition and morphology are the determining factors for critical stress (or peak maximum principal stress) during rupture. Plaque characteristics can be determined from numerical simulation for evaluation of its vulnerability [18-20]. In particular, patient-specific geometries can be reconstructed from MRI [21-27], and also with emphasis on the plaque rupture in the carotid artery due to high shear stress [28,29]. More importantly, a shift in paradigm occurs for the mechanism of fibrous cap rupture ranging from calcifications in arteries with lipid pools to cellular level microcalcifications in the fibrous cap. Effect of fibrous cap on plaque vulnerability has been widely investigated [30,31]. For a calcified plaque, the existence of some calcium core structural configurations is hypothesized to play a critical role in plaque rupture [32-34]. However, studies are limited to micro-calcification spots embedded in fibrous caps. The realistic calcification structures are present in the lipid and in agglomerates of clusters as presented by Huang et al. who showed the effect of percent areas of calcification and lipids on maximum principal stress [35]. But such patient-specific studies lack morphological parameters on controlled stress models and restrict insights into plaque rupture.

To address the current limitations, we model a realistic calcified plaque using variation of mechanical properties such as maximum principal stress and deformation due to the effect of morphological changes by calcification composites. A number of pathological and clinical imaging studies suggest that plaque vulnerability is inversely correlated with fibrous cap thickness. In addition to the fibrous cap thickness, the calcification gap which is defined as the width between the fibrous cap and the agglomeration of calcium clusters in the lipid is studied for the first time. To verify our hypothesis that calcification plays an important role in plaque vulnerability, idealized morphological constituents are implemented at different configurations to correlate stress parameters with geometrical properties.

## 2. Methods

### 2.1 Plaque Composite Model

The construction of the constitutive model is such that the complex behaviour of stress on the plaque can be quantified and analyzed. We assess stress on a plaque that comprises four main tissue types: the lipid (*lp*), the fibrous cap (*fc*), the calcium agglomerate (*cag*), the non-diseased wall (*ndw*). The morphological configuration of these components is of critical importance in the quantification of plaque vulnerability. The properties of these tissues are variable and integration of these various components into a plaque structure produces different stress effects.

In calcified plaques, agglomeration of microcalcification clusters is aligned in a crescent within the lipid and acts as a buoyant support to the rupture of the fibrous cap. Calcification clusters may be eccentrically shaped or positioned distantly from the lumen such that higher stress or tension may be localized at the fibrous cap. This causes an increase in plaque vulnerability as the calcification configuration tends to shift all the stress onto a focal point.

**Figure 2:**
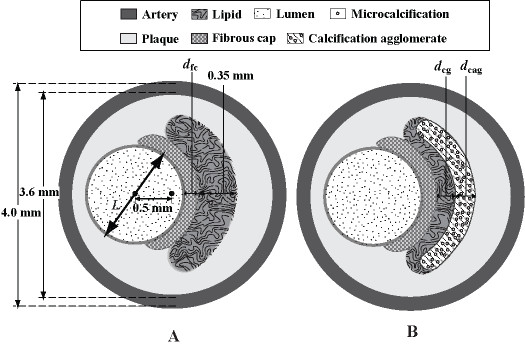
**Plaque configuration in atherosclerotic artery**. The presence of the fibrous cap and lipid within a diseased artery causes the protrusion of the arterial wall into the lumen. A: The morphological configuration of these plaque composites is idealized schematically to facilitate geometrical modeling for validation. B: The definition of a calcification gap is based on distance between the calcification agglomerate and the fibrous cap.

**Figure 3:**
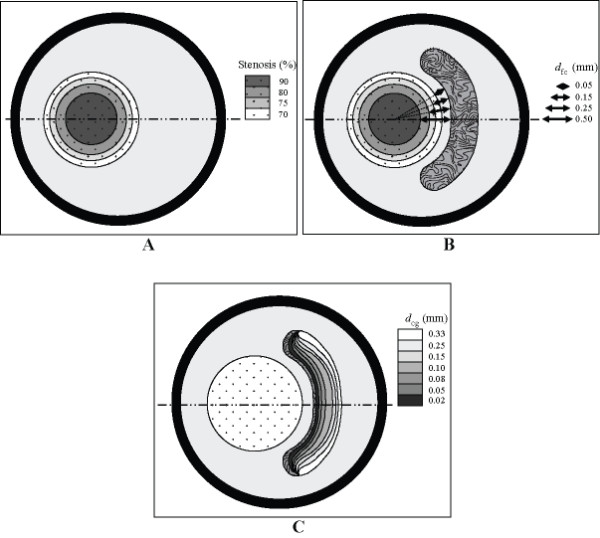
**Design of models based on varying geometrical configurations**. For model sets A and B (that are used for numerical simulation validation), we implement **t**he two sets of fibrous plaque geometries pertain to one of a constant material construction and one with a composite matrix of fibrous plaque and constant lipid core. Stenosis at 70, 75, 80 and 90% are modeled. As the position of lipid core (of constant thickness of 0.35 mm) remains consistent, the stenotic reduction results in thinning of fibrous cap. For model set C, we implement a set of calcified plaques which comprises of a varying calcification gap *d_cg _*(from 0.02 to 0.33 mm).

We model elastic behaviour of its composites by using stress-plane analysis on an idealistic model (Figure 2). The peripheral arterial internal diameter (3.6 mm) and external diameter (4.0 mm) are fixed. Fibrous material from plaque occupies the interior wall of an artery such that a lumen of varying size is formed. The lumen is modelled with an eccentricity of 0.5 mm with respect to arterial centre, and with varying lumen diameter *L *that corresponds to percentage of stenoses. The fibrous cap is assumed to be of the same material constitution as the plaque material. For set of models with lipids, this subintimal substance is constructed by extending a 140^o ^crescent with thickness of 0.35 mm (Figure 3A). The calcification gap refers to a lipid gap between fibrous cap and calcification agglomerate (Figure 3B). Plaque morphology is based on fibrous cap width *d_fc_*, thickness of calcification agglomerate *d_cag_*, and calcification gap *d_cg_*.

### 2.2 Plaque Rupture Mechanics

Anisotropic modelling of atherosclerotic vessel can be implemented to probe into plaque vulnerability issue [30,36]. A two-dimensional modelling platform for calibrating the extent of plaque rupture is based on mechanical parameters governing the atherosclerotic configuration. Three-dimensional analyses have also been prepared to justify the accuracy of the results based on the plane analyses of patient-specific case studies [18,20,21,31,33,37,38] that were previously investigated. Some studies of plaque mechanics examine arterial wall bending along the longitudinal axis since it has been shown that repetitive bending causes strain on an atherosclerotic plaque resulting in rupture [39].

Plaque rupture is dependent on biomechanical events acting on the fibrous cap such as hemodynamic shear stresses [40], turbulent pressure fluctuations [41], cyclic variation of intraluminal pressure and maximum principal stress by the pulsatile blood pressure [30,42]. In particular, large eccentric lipid cores are of mechanical disadvantage since circumferential tensile stresses are configured in such a way that fibrous caps have a tendency to rupture most of the time [36]. This gives rise to the relationship between plaque rupture and the critical stress acting on the fibrous cap.

Autopsies of patients that are diagnosed of cardiac ischemia showed that the level of macrophages is high, smooth muscle cells are reduced, the proportion of crescentic acellular mass for a lipid core is significant, and the fibrous cap is thin [42-45]. For plaque rupture, 65 μm thickness with an infiltrate of macrophages is defined as the threshold after histological analysis [46]. This can give guidance to critical risk analysis of plaque condition.

### 2.3 Design of Plaque Models

Idealized plane models of the longitudinal atherosclerotic arteries are implemented to study effects of stenotic severity on circumferential stress on plaque. One set pertains to stenosis based on a homogenous wall material while the other set is based on plaque with a lipid core where the constitutive model is taken to be non-homogenous, anisotropic, and elastic. To numerically simulate this type of plaque-vessel, all plaque constituents are assigned with the physiological mechanical properties.

For validation, we implement a non-calcified plaque structural configuration. We have two subsets of models that pertain to plaques with and without the lipid core in Figures 3A and 3B respectively. Then, we proceed to examine the effects of fibrous cap thickness *d_fc _*and width of calcification gap *d_cg _*on the stress levels that pertain to the plaque. Varying fibrous cap thickness *d_fc _*from 0.05 to 0.5 mm is implemented. We hypothesize that calcification plays an important role in plaque vulnerability assessment, and therefore the calcification agglomerate is modeled as a 140^o ^crescent of variable thickness *d_cag _*and positioned within the lipid. We design idealistic models for analysis of calcification structural variation which relates to calcification gap *d_cg_*, ranging from 0.05 to 0.33 mm (Figure 3C).

The following parameters are used in a plane-stress model: Young modulus (*E*) in circumferential (*θ*) and radial (*r*) directions, *ν_rθ _*and *ν_rz _*that are the Poisson ratios in *r-θ *and *θ-z *planes respectively, as well as *G_rθ _*that is the shear modulus in *r-θ *plane.

The elastic mechanical property of the calcification agglomerate is established based on defined percentages of fibrous plaque tissue (*ft*), lipid core (*lc*) and calcium (*Ca*):

(1) $$\begin{array}{c} E_{i}^{cag}=\alpha E_{i}^{ft}+\beta E_{i}^{lc}+\gamma E_{i}^{Ca},\\ G_{r\theta }^{cag}=\alpha G_{r\theta }^{ft}+\beta G_{r\theta }^{lc}+\gamma G_{r\theta }^{Ca}, \end{array}$$

where *i *denotes *r *and *θ *represents radial and circumferential orientations respectively. The percentage of compositions *α, β*, and *γ *corresponds to fibrous tissue, lipid and calcium, respectively. The Young modulus $E_{i}^{cag}$ and shear modulus $G_{r\theta }^{cag}$ are based on a linear combination of $E_{i}^{j}$ and $G_{r\theta }^{j}$ that pertains to component *j = ft, lc*, and *Ca*.

The material properties of the plaque constituents are consolidated from Loree et al. [30] and Holzapfel et al. [18] in Table 1. In our study, we assumed a combination of fibrous tissue (*α *= 5%), lipid (*β *= 20%) and calcium (*γ *= 75%) as components for a homogenous calcification agglomerate.

**Table 1:** Material properties for plaque constituents.

Parameter	Artery (ndw)	Fibrous tissue (ft)	Lipid (lp)	Micro-calcium (Ca)	Calcification agglomerate (cag)
*E_r _*(kPa)	10	50	1	12,600	9452.7
*E_θ _*(kPa)	100	1000	1	12,600	9500.2
*G_rθ _*(kPa)	50	500	1	12,600	9475.2
*ν_rθ_*	0.01	0.01	0.01	0.01	0.01
*ν_rz_*	0.27	0.27	0.27	0.27	0.27

The Young modulus (*E_r_, E_θ_*), Poison ratios (*G_rθ_*) and shear modulus (*ν_rθ_, ν_rz_*) are presented for the plaque, non-diseased wall of artery, and lipid. The data are revised from Loree et al. [30] and Holzapfel et al. [18] Fibrous tissue at *α *= 5%, lipid at *β *= 20% and calcium at *γ *= 75% pertain to the homogenous calcification agglomerate.

### 2.4 Two-dimensional Finite Element Method Validation

**Figure 4:**
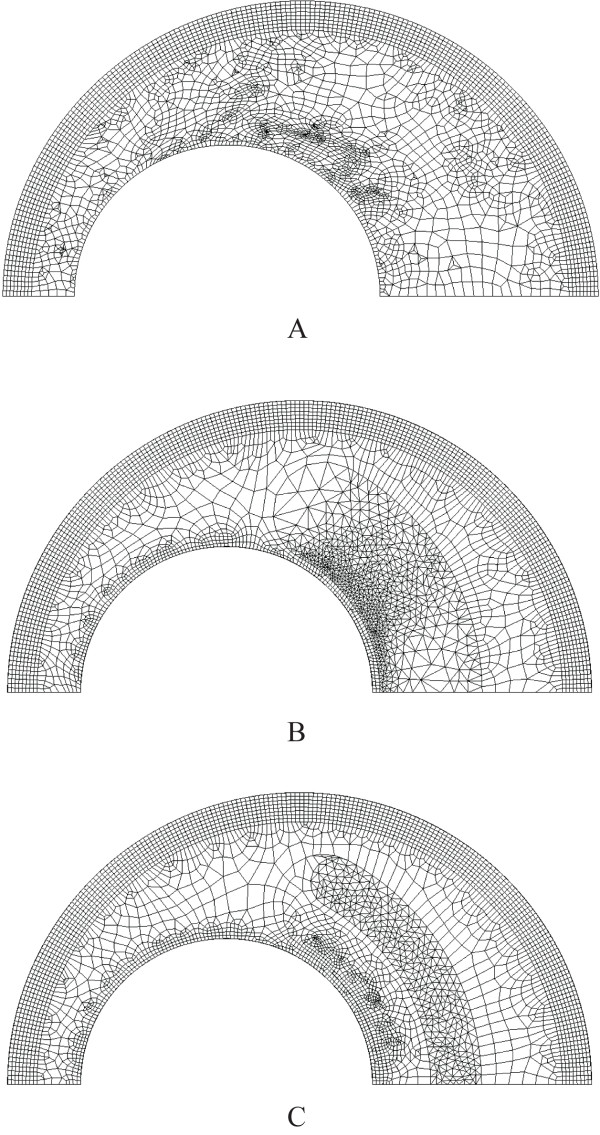
**Mesh modelling of plaque model based on different configuration of lipid pool and calcification agglomerate at 70% stenosis**. A: For plaque with no lipid pool (70% stenosis), the geometrical modeling uses *N *= 12928 for plane-strain elements. B: For plaque with constant lipid pool (of thickness 0.35 mm), *N *= 12712 elements. C: For plaque with constant lipid pool and calcification agglomerate (*d_cg _*= 0.175 mm), *N *= 12123 elements.

Finite element method (FEM) is performed using ANSYS^® ^finite element program to relate the stress distribution within plaque with a luminal pressure *P *of 14.6 kPa. The finite element meshes are based on tetrahedral elements with minimal skewness. The axial strain is of the order of vessel dimension and negligible with respect to the circumferential and principal strain, and therefore the analysis is based on plane-stress models. Due to the symmetry of the vessel, a half model is implemented to reduce computational costs of modelling stress. The symmetry condition is applied at the half-vessel that lies on a symmetry line. Adaptive meshing for each component of the plaque is performed to increase the mesh resolution at regions where high strain energies are localized. Such non-homogenous distribution of mesh elements will improve accuracy of the numerical solution. Different grid densities are applied for the artery, fibrous plaque, lipid and calcification agglomerate as a variation of strain energies pertain to these elastic materials (Figure 4).

As atherosclerosis is a complex process, multiple parameters are required to accurately model plaque vulnerability. As a prerequisite, it is useful to conduct this preliminary analysis based on a simplified version of the model in order to identify the correlations between maximum principal stress, maximum deformation, fibrous cap thickness and calcification gap. Prior to these numerical experiments, a validation is performed against research study by Loree et al. [30] based on idealised atherosclerotic plaque configuration using planar stress analysis.

**Figure 5:**
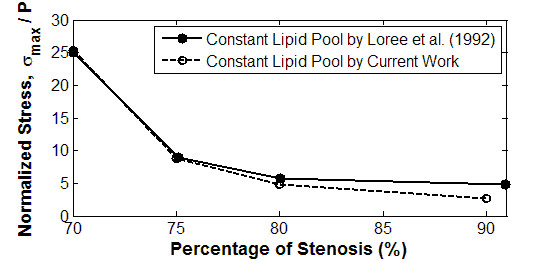
**Relationship between circumferential stress and stenosis**. The graph of the circumferential stress versus degree of stenosis is presented for plaque with or without lipid pool. The peak circumferential stress is normalized with respect to the luminal pressure (σ_max_/P). This can give an indication of the accuracy of simulation analysis using results by Loree et al. [30] as a form of validation

Figure 5 presents the effect of fibrous cap thickness on peak circumferential stress. For cases with constant lipid, fibrous cap thickness reduces with decreasing stenosis. In addition, when there is a constant lipid inside the plaque, the level of stress tends to be strongly influenced by the thickness of fibrous cap. Our simulation results agree well with validation data. We also deduce that since the fibrous cap thickness correlates to plaque stability, it is an important parameter when determining plaque vulnerability.

### 2.5 Three-dimensional Computational Fluid Dynamics Modelling

To analyze the structure of the plaque components, numerical simulation is applied to illustrate the variation of mechanical properties due to the effect of changes by the lipid core and its agglomerate of microcalcification. Modelling of the human atherosclerotic artery with varying degrees of lipid core elasticity, fibrous cap thickness and calcification gap, which is defined as the distance between the fibrous cap and calcification agglomerate, form the basis of our rupture analysis.

#### 2.5.1 Geometry Reconstruction and Meshing

Tada et al. performed modelling of healthy carotid bifurcation based on an idealistic geometry [47]. Key dimensions of this artery are presented in Table 2. For their geometry, a region of sinus is included as it is the common feature found in carotid bifurcation. Our model was based on a size scale of 1:2.775 when compared with the two-dimensional verification model. However, this will not affect the structural analysis if all our structural parameters are varied at the same specific ratios to achieve physiological similitude.

**Table 2:** Geometrical properties for carotid bifurcation.

Location of Carotid Bifurcation	Dimensions
CCA internal diameter	0.01 m
Maximum sinus internal diameter	0.23 m
ICA internal diameter	0.007 m
ECA internal diameter	0.0065 m
ICA bifurcation angle	25°
ECA bifurcation angle	25°

The dimensions for a generic carotid bifurcation are presented based on the model by Tada et al.^50 ^to be used in CFD simulation.

**Figure 6:**
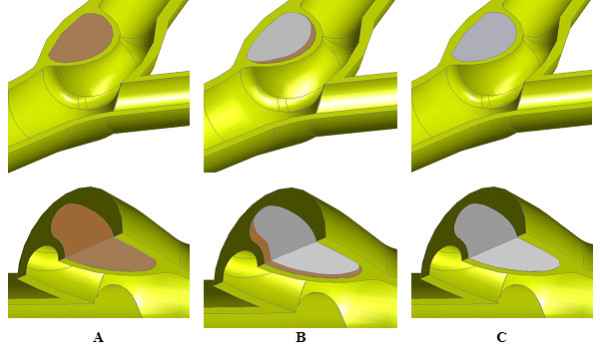
**Three-dimensional isometric view of the plaque in a carotid bifurcation**. The plaque-arterial configuration can be non-calcified plaque, mixed type of plaque and calcified plaque. Here, the lipid height and width is specified as 6.714 mm and 3.298 mm respectively, with the fibrous cap at 0.500 mm thick.

For a diseased carotid bifurcation, the location of the plaque is assumed to be located on the outer wall of the internal carotid artery (ICA) sinus in order to study the effect of the stenosis to the flow as well as the mechanical stress occurs. A three-dimensional crescent structure was incorporated into the sinus of the carotid bifurcation to simulate the presence of plaque. We note that plaques can be characterized into three types based on the component with it: non-calcified plaque, partially and fully calcified plaque (see Figure 6A, B and 6C respectively). Fibrous tissue (*α*), lipid (*β*) and calcium (*γ*) pertain to the homogenous calcification agglomerate at *α *= 5%, *β *= 20% and *γ *= 75% as components for a homogenous calcification agglomerate.

We can implement an anisotropic modelling of the atherosclerotic vessel to probe into the plaque vulnerability issue. We present a three-dimensional modelling platform for calibrating the extent of plaque rupture based on mechanical parameters governing the atherosclerotic configuration. Then analyses of some sample case will be prepared to justify the accuracy of the results based on the plane analyses. Some studies of plaque mechanics examine arterial wall bending along the longitudinal axis since it has been shown that repetitive bending causes strain on an atherosclerotic plaque resulting in rupture [39].

**Figure 7:**
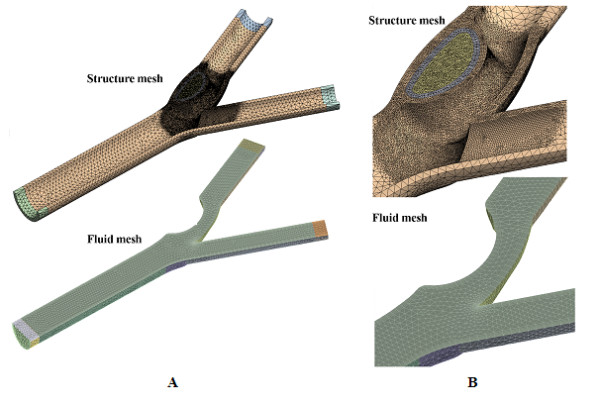
**Meshing of calcified carotid bifurcation**. The isometric view of the mesh for entire length of carotid bifurcation and a zoom-in view of the calcified plaque gives an indication of the mesh resolution required for blood-plaque interaction simulation.

Figure 7 shows example of computational grid used in this research. Figure 7A illustrates the mesh of the full calcified carotid bifurcation. Figure 7B reveals the resolution of the calcification that is required for the simulation. In each case, the mesh will be slightly different due to the degree of stenosis. The mesh used consisting of three-dimensional tetrahedral and prism was generated in CFX-mesh software. Grid independence analysis was performed at 3 different mesh refinement levels for the structure domain: Coarse (130,000 elements), Medium (340,000 elements) and fine (1,500,000 elements). For the fluid domain, the mesh refinement levels are: Coarse (110,000 elements), Medium (300,000 elements) and fine (1,000,000 elements). For both the structure and fluid domain, only 2% of dissimilitude between the fine and medium mesh was observed. Therefore, it is concluded that the fine mesh can be used to obtain grid independent results. All results obtained and discussed in the discussion section are the solution of computation performed with the fine mesh.

#### 2.5.2 Details of Blood-Vessel-Plaque Simulation

Partitioned approach was used to implement FSI capability in the ANSYS^® ^software package. The coupling is performed between ANSYS and CFX. The coupling of the two solvers is performed many times per time step until convergence of interface variables (displacements and pressure) is reached. At each coupling loop, calculation of blood flow is initiated. Then calculated pressure field is transferred and used as applied force in ANSYS in order to calculate deformation of the artery. The tolerance for the interface variables is 1E-4. The blood flow is modelled a laminar since the highest Reynolds number, even with high degree of stenosis, is approximately about 1000 which is still in laminar region. Time step size is set to 0.015 s. the results is obtained at the 4^th ^cycle to get rid of effects from initial conditions.

**Figure 8:**
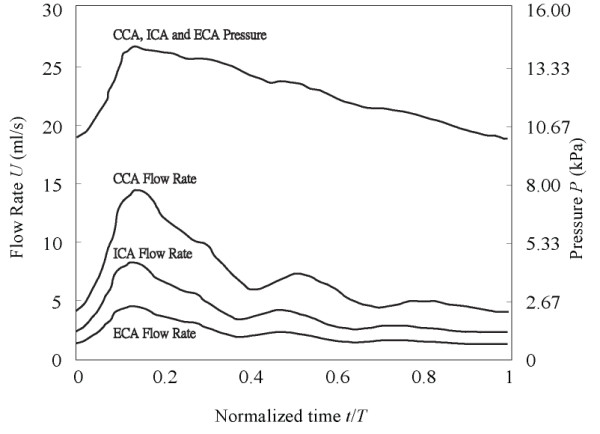
**Patient-specific pressure waveform used in simulation**. Patient-specific pressure waveform for the fluid simulation within the carotid bifurcation shows a maximum of 107 mmHg used. This waveform is imposed at the entrance of the artery.

In solid domain, each end of the artery (CCA, ICA, ECA) are modelled as fixed supports while symmetry condition is assumed at the plane of the bifurcation. In fluid domain, boundary condition at inlet is specified as time-varying constant [47] while outlet boundary conditions at the end of ICA and ECA are set as time-varying mass flow rate as shown in Figure 8. In addition, no slip condition is specified at artery wall. The wall is assumed to be smooth. In this work, arterial wall is modelled as a Hookean and isotropic material for computational simplicity [48,49]. The blood properties are also simplified and thus modelled as Newtonian fluid. Both blood and artery properties are shown in Table 3.

**Table 3:** Material properties for artery and blood constituents.

Artery	
Young modulus (Pa)	5.00E+05
Poisson ratio	0.5
**Blood**	

Density (kg m^3^)	1050
Viscosity (Pa s)	3.50E-03

The Young modulus and Poison ratio for artery as well as density and viscosity for blood are presented for the blood-vessel interaction simulation.

## 3. Results and Discussion

### 3.1 Two-dimensional Structural Modelling

Subintimal plaque structures such as fibrous cap thickness play an important role in plaque stress distribution. Here, we analyze the pathological fracture caused by the increases of stress on plaque. We have, in addition to this parameter, the calcification gap (which is defined as the width of the lipid layer sandwiched between the calcification agglomerate and the fibrous cap) as another variable. Due to a matrix of different elastic materials in the composition, stress concentrations vary throughout the structure [50,51]. Therefore, it is of interest to simulate how the variable morphological configurations affect the stress levels on the plaque which can cause fracture. Then, sensitivity studies on effects of lipid elasticity and fibrous cap thickness in the case of a constant lipid core on maximum principal stress and deformation are presented.

**Figure 9:**
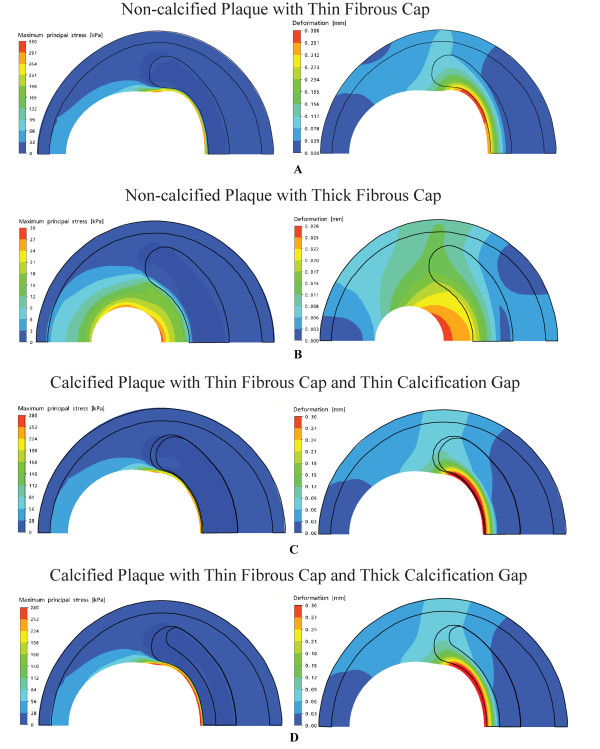
**Finite element analysis of plaque models at two-dimensional cross-sections and with different fibrous cap thickness and calcification gap**. Peak principal stress and deformation plots pertain to a constant lipid pool (*E*_lp _= 1 kPa) of fixed thickness (0.35 mm), and with varying fibrous cap thickness *d_fc _*and calcification gap *d_cg_*. A: For *d_fc _*= 0.05 mm, the critical stress *σ*_cr _and maximum deformation *D*_max _are 331 kPa and 0.390 mm respectively. B: For *d_fc _*= 0.48 mm, *σ*_cr _= 28.7 kPa and *D*_max _= 0.0281 mm. The geometrical outlines of the plaque composites for A and B show the structural difference in the deformed plaque due to applied stress. C: For *d_cg _*= 0.02 mm and *d_fc _*= 0.05 mm, *σ*_cr _= 282 kPa and *D*_max _= 0.290 mm. D: For *d_cg _*= 0.175 mm and *d_fc _*= 0.05 mm, *σ*_cr _= 326 kPa and *D*_max _= 0.370 mm.

Results for plaque models at 70% and 90% stenosis and with a constant lipid pool (*E*_lp _= 1 kPa) are illustrated by Figures 9A and 9B. Analysis of the different plaque models with lipid cores of fixed size (0.35 mm) shows the effect of fibrous cap thickness *d_fc _*on maximum principle stress and deformation. Multiple numerical simulation models based on the variation of *d_fc _*and *E*_lp _is performed to characterize critical stress and maximum deformation levels. The sensitivity of the mechanical stress properties to the lipid core elasticity and fibrous cap thickness can be presented with response curves that provide the interaction between different mechanical properties of the plaque material. This can give us an insight into the morphological effect of plaque constituents on maximum stress levels.

Figures 9C and 9D are simulated models with a constant lipid cores whose Young Modulus is set as *E*_lp _= 1 kPa and a calcification agglomerate that has Young Modulus *E*_cag _based on *α *= 5%, *β *= 20% and *γ *= 75% (refer to Table 1). The changes in these mechanical properties can be graphically presented when calcium clusters are present. Variation of calcification gap *d_cg _*is presented to show its effect on peak principal stress and maximum deformation. Modelling calcified plaque with agglomerate at varying calcification gaps gives the response of maximum principal stress and deformation based on the influence of calcium clusters. This mechanical entity affects structural integrity of the overall plaque content, and plays a major role in plaque vulnerability.

### 3.2 Three-dimensional Fluid-Structure Interaction Modelling

**Figure 10:**
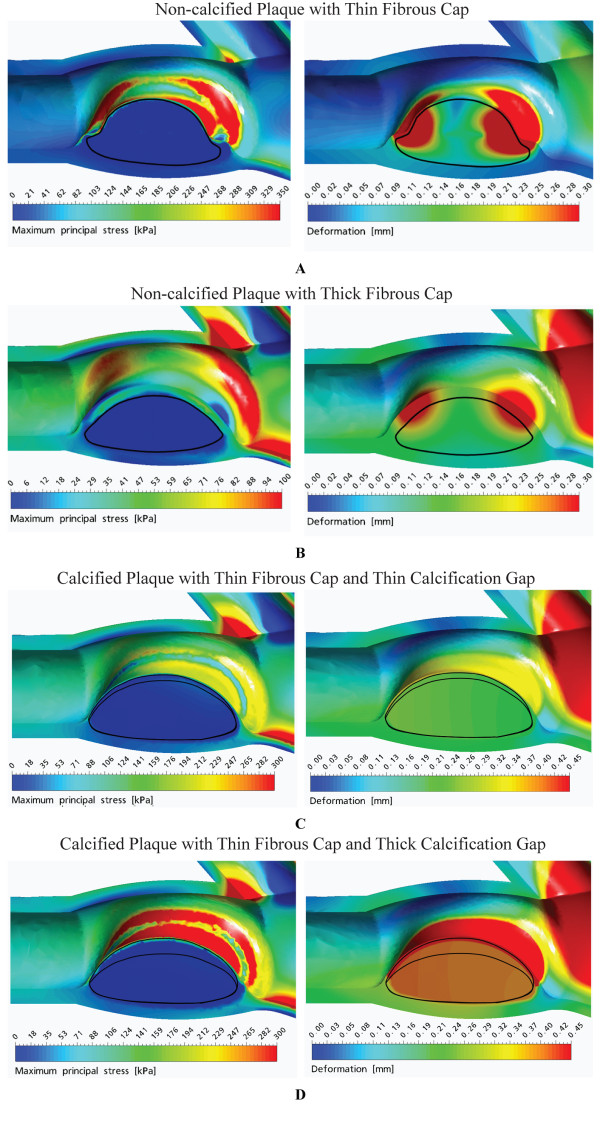
**Fluid-structural interaction analysis of three-dimensional plaque models based on a longitudinal orientation and with different fibrous cap thickness and calcification gap**. Peak principal stress and deformation plots pertain to a constant lipid pool (*E*_lp _= 1 kPa) of fixed size (0.35 mm), and with a specific fibrous cap thickness *d_fc _*and calcification gap *d_cg_*. A: For *d_fc _*= 0.05 mm, the critical stress *σ*_cr _and maximum deformation *D*_max _are 350 kPa and 0.421 mm respectively. B: For *d_fc _*= 0.05 mm, *σ*_cr _= 95.6 kPa and *D*_max _= 0.293 mm. C: For *d_cg _*= 0.1 mm and *d_fc _*= 0.05 mm, *σ*_cr _= 258 kPa and *D*_max _= 0.352 mm. D: For *d_cg _*= 0.3 mm and *d_fc _*= 0.05 mm, *σ*_cr _= 314 kPa and *D*_max _= 0.467 mm.

Figure 10 are simulated three-dimensional models with a constant lipid cores at *E*_lp _= 1 kPa and a calcification agglomerate where *E*_cag _is based on *α *= 5%, *β *= 20% and *γ *= 75% (refer to Table 1). We extract the maximum principal stress and deformation contour plots for the carotid bifurcation along its longitudinal axis as it is more easily visible to observe these mechanical property variations along the fibrous cap.

The three-dimensional plaque models at 90% stenosis under the effect of different fibrous cap configurations are illustrated by Figures 10A and 10B. The different plaque models with lipid cores of fixed size is effected and the influence of fibrous cap thickness *d_fc _*on maximum principle stress and deformation is demonstrated to be similar to the trend shown by the two-dimensional structural analysis, whereby increment in the fibrous cap thickness *d_fc _*results in a reduction of critical stress and maximum deformation.

Figures 10C and 10D are simulated blood-plaque-vessel models in which variation of calcification gap *d_cg _*is presented to show its effect on peak principal stress and maximum deformation. Here, increment of *d_cg _*results in an increase of these two mechanical properties.

### 3.3 Response of Maximum Principal Stress and Deformation to Plaque Elasticity and Structural Variation

Response curves for stress and deformation versus plaque composite elasticity and fibrous cap thickness are plotted. Both maximum principal stress and deformation have negative correlation with the fibrous cap thickness and Young modulus of plaque composites. This leads to the suggestion that the change of stress with respect to Young modulus of lipid core or calcification agglomerate and fibrous cap thickness tends to follow the same variation as deformation. Calcification gap and maximum deformation thresholds are established based on critical stress threshold for plaque rupture.

#### 3.3.1 Two-dimensional Structural Analysis

**Figure 11:**
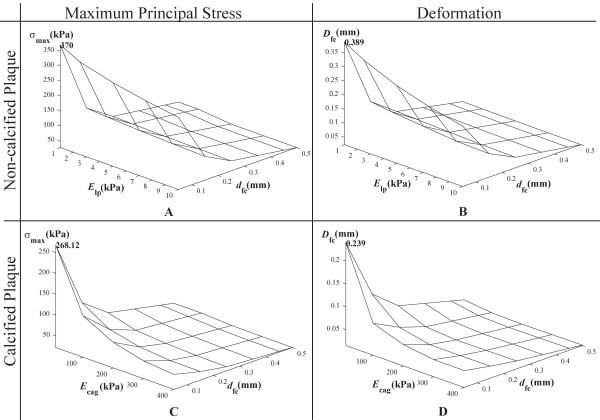
**Maximum principal stress and deformation based on elasticity of two-dimensional plaque composite and fibrous cap thickness for non-calcified and calcified plaque**. A: For a two-dimensional non-calcified plaque, graph of the maximum principal stress *σ*_max _versus Young modulus *E*_lp _and fibrous cap thickness *d*_fc _shows that critical stress is 370 kPa. B: For a two-dimensional calcified plaque at *d*_cg _= 0.02 mm, the fibrous tissue, lipid and calcium structures are present at various concentrations in the calcification agglomerate such that its Young modulus *E*_cag _varies from 10 to 400 kPa. The plot of *σ*_max _versus *E*_cag _and *d*_fc _shows that critical stress is 268.12 kPa. C: For a two-dimensional non-calcified plaque, graph of peak deformation *D*_fc _versus *E*_lp _and *d*_fc _shows that maximum deformation *D*_max _is 0.389 mm. D: For a two-dimensional calcified plaque, graph of *D*_fc _versus *E*_cag _and *d*_fc _gives *D*_max _= 0.239 mm.

Stress response curve for maximum principal stress *σ*_max _versus Young modulus of lipid *E*_lp _and fibrous cap size *d*_fc _shows that the peak maximum principal stress or critical stress *σ*_cr _is 370 kPa, which corresponds to the highest plaque vulnerability, is achieved where the plaque has lipid core with the highest elasticity and the thinnest fibrous cap (Figure 11A). Stress levels of calcified plaque (where *E*_lp _= 1 kPa, *d*_cg _= 0.02 mm and *E*_cag _ranges from 10 to 400 kPa) demonstrates the same correlation with plaque composite elasticity and fibrous cap thickness (Figure 11C). Critical stress for a calcified plaque (*σ*_cr _= 268.12 kPa) is lower than that of a non-calcified one. In general, the stress levels of the calcified plaque are lower than a non-calcified one.

Effect of *E*_lp _and *d*_fc _on the cap deformation *D*_fc _is presented (Figure 11B). The peak deformation *D*_max _at 0.389 mm or 389 μm corresponds to the lower limit of the range that pertains to lipid core Young modulus and fibrous cap thickness. With calcification, *D*_max _is reduced to 0.239 mm (Figure 11D). The overall deformation is generally lower than that for the non-calcified plaque. The deformations are an order of magnitude higher than the fibrous cap for plaque rupture.

#### 3.3.2 Three-dimensional Fluid-Structural Analysis

**Figure 12:**
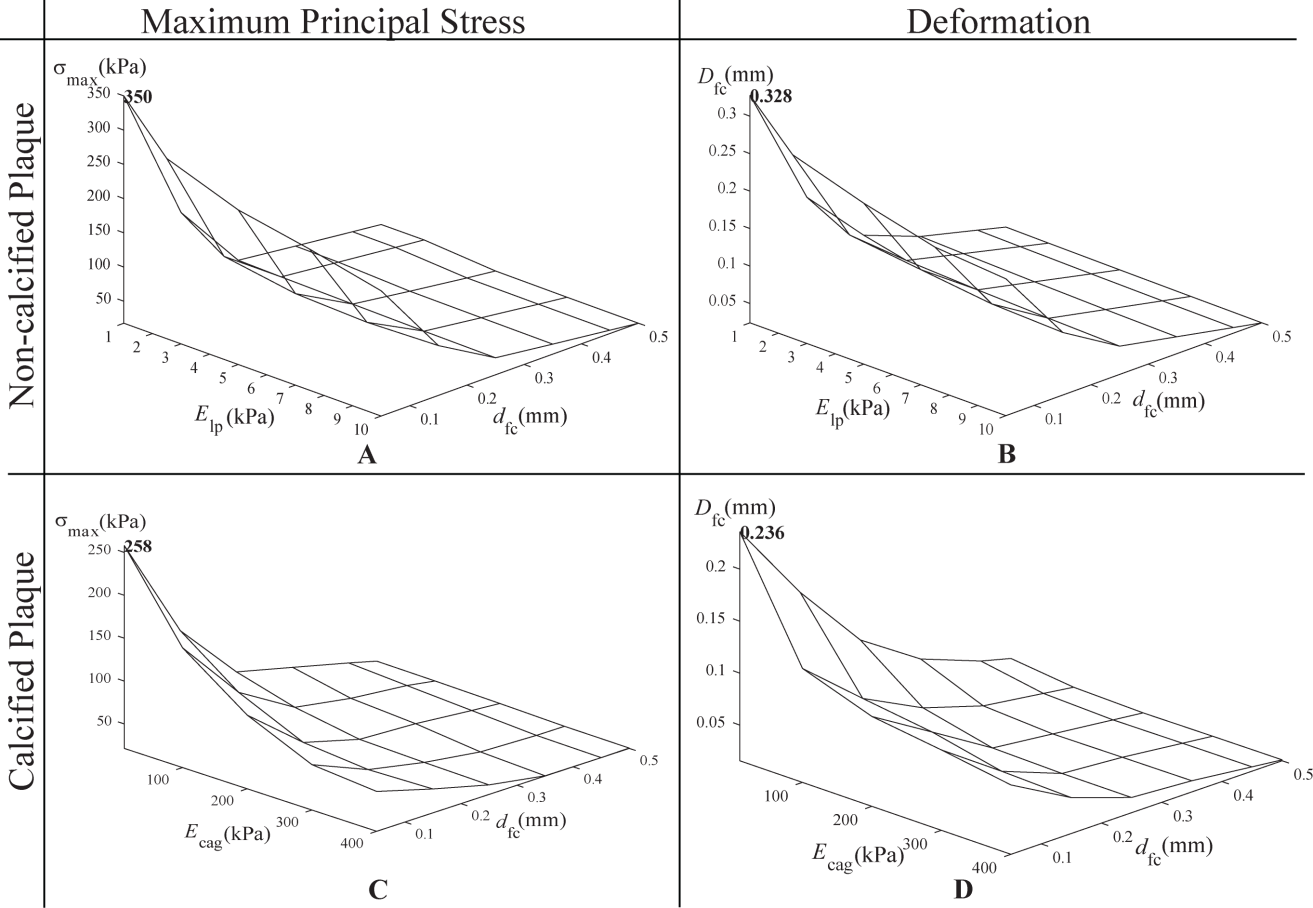
**Maximum principal stress and deformation based on elasticity of three-dimensional plaque composite and fibrous cap thickness based on non-calcified and calcified plaque**. A: For a three-dimensional non-calcified plaque, graph of the maximum principal stress *σ*_max _versus Young modulus *E*_lp _and fibrous cap thickness *d*_fc _shows that critical stress is 350 kPa. B: For a three-dimensional calcified plaque at *d*_cg _= 0.02 mm, the plot of *σ*_max _versus *E*_cag _and *d*_fc _shows that critical stress is 258 kPa. C: For a three-dimensional non-calcified plaque, graph of peak deformation *D*_fc _versus *E*_lp _and *d*_fc _shows that maximum deformation *D*_max _is 0.328 mm. D: For a three-dimensional calcified plaque, graph of *D*_fc _versus *E*_cag _and *d*_fc _gives *D*_max _= 0.236 mm.

We note the improvement in smoothness of the surface curve variation of the graph based on three-dimensional fluid-plaque simulation in the atherosclerotic carotid bifurcation. Based on Critical stress for a non-calcified plaque at *σ*_cr _= 350 kPa (shown in Figure 12A) is higher than that of a calcified one with *d*_cg _= 0.1 mm at *σ*_cr _= 258 kPa (shown in Figure 12C). *D*_max _at 0.328 mm corresponds to the maximum deformation for non-calcified plaque (Figure 12B). With calcification, *D*_max _is reduced to 0.236 mm (Figure 12D).

Typically, the simulation results follow the same trend as that of the two-dimensional plaque structural analysis. We see a drop in value of the blood-vessel interaction model when compared based on the two-dimensional structural analysis. However, the critical stress and maximum deformation follows a more accurate trend due to the realism of the blood-plaque configuration being modeled. It may be worthwhile highlighting that the two-dimensional analysis can serve as a preliminary verification of the three-dimensional results.

### 3.4 Response of Critical Stress and Maximum Deformation to Plaque Structural Variation

Relationship between calcification gap and maximum principal stress is based on effect of stress distribution on fibrous cap having *d*_cg _varied from 0 to 0.25 mm and with *E*_lp _= 1 kPa and *E*_cag _= 100 kPa. Plaque rupture occurs when stress levels exceed a 300 kPa threshold as determined by Lendon et al. [52] and Vengrenyuk et al. [32] This stress threshold determines that based on the morphological condition that we assumed in our model and for a threshold calcification gap, plaque fracture will occur. It is worthwhile mentioning that it should not be assumed that all plaques fracture at this value [36]. However, this value can be used as a guide in our analysis.

#### 3.4.1 Two-dimensional Structural Analysis

For the non-calcified plaque with the same fibrous cap thickness, stress level can reach as high as near 370 kPa. But presence of calcification agglomerate at sufficiently low calcification gap can lower stress levels to below 370 kPa and prevent plaque rupture which may occur at 300 kPa. Since fibrous cap thinness threshold for rupture is 0.065 mm, we implement the case of a fibrous cap as thin as 0.05 mm as a limiting example. The calcification gap is specified as 0.02 mm as consistent with Figure 11.

**Figure 13:**
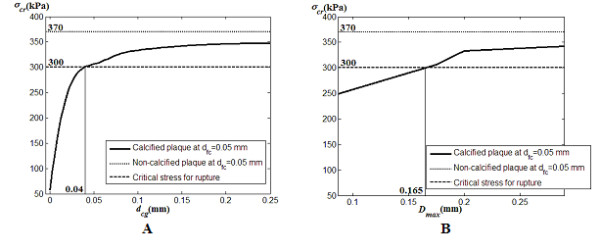
**Critical stress on fibrous cap with respect to width of calcification gap and maximum deformation on fibrous cap for two-dimensional plaque-rupture analysis**. A: The plot of critical stress *σ*_cr _versus width of calcification gap *d*_cg _reflects the decrease in plaque vulnerability for increasing occupancy of the calcification agglomerate (which is inversely correlated to *d*_cg_). For *d*_fc _at 0.05 mm as a conservative setting, a calcification gap value of > 0.04 mm causes stress levels to exceed 300 kPa and cause plaque rupture. B: Critical stress *σ*_cr _versus maximum deformation *D*_max _of fibrous cap for *d*_fc _at 0.05 mm demonstrates that *σ*_cr _becomes lower as *d*_cg _minimizes the deformation. Here, *D*_max _> 0.165 mm causes plaque rupture.

Based on calcified plaque with fibrous cap thickness *d*_fc _at 0.05 mm, the relationship between calcification gap *d*_cg _and peak maximum principal stress or critical stress *σ*_cr _is presented (Figure 13A). As calcification gap increase, the critical stress tends to converge to a stable levelling of peak maximum principal stress. The plaque is stabilized when the calcification gap is less than 0.04 mm based on the assumed plaque configuration. The critical stress *σ*_cr _has a positive correlation with maximum fibrous cap deformation *D*_max _(Figure 13B). This is due to the correlation that exists for the calcification gap with the maximum deformation. For *D*_max _> 165 μm, which is 3.3 times the fibrous cap thickness (0.05 mm), stress levels exceed 300 kPa.

#### 3.4.2 Three-dimensional Fluid-Structural Analysis

**Figure 14:**
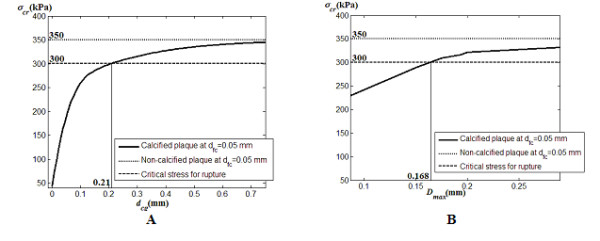
**Critical stress on fibrous cap with respect to width of calcification gap and maximum deformation on fibrous cap for three-dimensional plaque rupture analysis**. A: The plot of critical stress *σ*_cr _versus width of calcification gap *d*_cg _shows that calcification gap value of > 0.21 mm causes stress levels to exceed 300 kPa and cause plaque rupture. B: Critical stress *σ*_cr _versus maximum deformation *D*_max _of fibrous cap for *d*_fc _at 0.05 mm demonstrates that *D*_max _> 0.168 mm causes plaque rupture.

As shown in Figure 14, the same trend follows for the three-dimensional model with calcified plaque whose calcification gap is specified at 0.1 mm. Here, Figure 14A illustrates the relationship between calcification gap *d*_cg _and critical stress *σ*_cr_, and Figure 14B correlates the critical stress *σ*_cr _with maximum fibrous cap deformation *D*_max_. We note a slight reduction in critical stress below the 350 kPa threshold value. The limiting calcification gap occurs at *d*_cg _= 0.21 mm before plaque rupture takes place for critical stress at 300 kPa. It is to be noted that a larger calcification gap is presented as compared to the results of the two-dimensional model occurs due to the implementation of an artery-plaque structure that is 2.775 times larger in size.

The maximum deformation also assumes the same trend that is based on the two-dimensional structural stress analysis. For a larger artery being configured, and implementation of a carotid bifurcation model, we see a reduction in terms of value for critical stress. The maximum deformation is observed to be approximately the same at *D*_max _> 168 μm for the stress levels to exceed 300 kPa.

## 4. Conclusion

Medical imaging modalities are able to characterize the atherosclerotic plaque in terms of their morphological and mechanical properties. Non-invasive imaging techniques not only identify flow-limiting vascular stenosis, but also detect calcified and non-calcified plaque, measure atherosclerotic plaque burden and its response to treatment, and differentiate stable plaques from those which tend to rupture [4,5]. However, the prediction of high-risk plaque rupture still requires a numerical simulation framework for verification due to the complex matrix of different material composites. This can form the basis for determining adverse cardiovascular events that have exceeded the threshold for rupture.

Subintimal plaque structures such as the fibrous cap, calcification gap and lipid core play an important role in determining plaque rupture. For a non-calcified plaque with constant luminal area, the critical stress and peak deformation increase as the fibrous cap becomes thinner. On the contrary, these two mechanical effects lessen in the presence of calcification agglomerates. For a thin fibrous cap and a large calcification gap, the stress levels will be significant and results in high vulnerability of the plaque despite the fact that they may show angiographically insignificant. Therefore, the subintimal structure should be used as the basis for determining plaque vulnerability instead of information on stenotic severity that is based on medical image visualisation.

Macrocalcifications occupy part of the lipid pool and that the cellular and smaller calcifications are distinct from these macrocalcifications which forms another category. All natures of the calcifications may coexist in the lipid pool and are independent of one another. We made an assumption in the model that the microcalcifications are floating debris uniformly distributed in the lipid pool without adhesion to form larger macrocalcification structures. While this may not form the true composite in reality, the effect of calcification can still be modelled by this configuration.

Calcification clusters plays a major role in plaque rupture as demonstrated by structural analysis on a continuous calcification agglomerate structure. Some studies showed a negative effect on plaque vulnerability and demonstrated that stress induced by microcalcification in thin fibrous caps advances plaque rupture [32-34]. Others suggested that calcification stabilizes plaque [35,36]. Cellular calcification structures introduce a role in plaque vulnerability, and our study may be of interest to the analysis of calcification structure based on agglomerates of micro-calcium elements in plaque. In reality, calcium clusters are scattered in the form of a crescent shape within the lipid core. To examine the collective effect of these calcium clusters such as their distance from the fibrous cap, we assume a continuous calcification structure along the curvature of the artery with a layer of lipid volume in between. Our agglomerate model is a linear combination of microcalcification, fibrous plaque and lipid at specific percentages and assumed a uniform property based on this homogenous mixture, which may be adjusted depending on patient-specific density of calcium in plaque.

We arbitrarily assume the configuration of the computational models based on observation of the histologic images of partially calcified plaque. It represents a particular stage of calcified plaque development. The nature of analysis would remain the same even though this configuration is modified at a later stage of the development.

## Competing interests

The authors declare that they have no competing interests.

## Authors' contributions

KW carried out the computational studies, participated in the modelling work and drafted the manuscript. PT developed the simulations based on fluid-structure interaction, prepared the results for analysis and helped to draft the manuscript. SC participated in the finite element modelling and analysis. ZS participated in the design of the study and contributed to the medical imaging background of this manuscript. JT conceived the entire study, and participated in its design and coordination. All authors read and approved the final manuscript.

## Pre-publication history

The pre-publication history for this paper can be accessed here:

http://www.biomedcentral.com/1471-2261/12/7/prepub
